# Effect of CdO on the Structural and Spectroscopic Properties of Germanium–Tellurite Glass

**DOI:** 10.3390/ma18081739

**Published:** 2025-04-10

**Authors:** Iveth Viridiana García Amaya, David Alejandro Rodríguez Carvajal, Josefina Alvarado-Rivera, R. Lozada-Morales, Paula Cristina Santos-Munguía, Juan José Palafox Reyes, Pedro Hernández-Abril, Gloria Alicia Limón Reynosa, Ma. Elena Zayas

**Affiliations:** 1 Ingeniería en Geociencias, Unidad Hermosillo, Universidad Estatal de Sonora, Hermosillo 83100, SON, Mexico or david.rdgz.c@gmail.com (D.A.R.C.); paula.santos@ues.mx (P.C.S.-M.); 2 Departamento de Investigación en Física, Universidad de Sonora, Hermosillo 83000, SON, Mexico; 3 SECIHTI—CINVESTAV-Querétaro, Libramiento Norponiente 2000, Fracc. Real de Juriquilla, Querétaro 76230, QRO, Mexico; josefina.alvarado@cinvestav.mx; 4 Posgrado en Física Aplicada, Facultad de Ciencias Físico Matemáticas, Benemérita Universidad Autónoma de Puebla, Calle 14 Sur y Av. San Claudio, Col. Jardines de San Manuel, Puebla 72570, PUE, Mexico; rlozada@fcfm.buap.mx; 5 Departamento de Geología, Universidad de Sonora, Hermosillo 83000, SON, Mexico; juan.palafox@unison.mx; 6 Ingeniería Biomédica, Unidad Hermosillo, Universidad Estatal de Sonora, Hermosillo 83100, SON, Mexico; pedro.hernandez@ues.mx; 7 Departamento de Ciencias Químico-Bilógicas y Agropecuarias, Universidad de Sonora, Navojoa 85880, SON, Mexico; gloria.limon@unison.mx

**Keywords:** tellurite glasses, non-bridging oxygens, Raman, band gap, binding energy

## Abstract

New glasses in the xCdO-(90 − x)TeO_2_-10GeO_2_ system were obtained by the conventional melt-quenching process at 900 °C. The glasses were transparent to the naked eye. The diffraction patterns indicate that the samples were mostly amorphous, except for the CdO-rich glasses, in which the formation of nanocrystals of CdO and Cd_3_TeO_6_ were identified. Raman spectroscopy analysis of the samples displayed the existence of TeO_3_, TeO_3+1_, TeO_4_, and GeO_4_, structural units within the glass matrix. The optical band gap of the glass samples was determined by optical absorption spectroscopy using the Tauc method. Depending on the relative content of TeO_2_, their values varied in the range of 2.32–2.86 eV. The refractive index was obtained from the band gap values. The XPS measurements showed that Ge 3d, O 1s and Te 3d_3/2_, Te 3d_5/2_, Cd 3d_5/2_, and Cd 3d_3/2_ doublets shifted to higher binding energy values as the amount of TeO_2_ was increased. The binding energy values of the Te 3d doublet are related to the TeO_4_ and TeO_3_ groups.

## 1. Introduction

Research on glass matrices containing germanate and tellurate has received special attention in the past because it has been found that this type of glass, when hosting rare earth ions, has applications in the mid-infrared. This is because the reduction of the phonon of the host glass is a condition for achieving high-efficiency mid-infrared luminescence. It is here that these glasses present interesting applications in lasers, which are useful in surgery, atmospheric pollution control, military uses, and remote sensing, among other applications [1].

On the other hand, tellurite glasses in the field of optics are of great interest because are materials for applications in optical waveguides, Raman amplifiers, and third-order harmonic generation [2,3,4]. Moreover, they have become relevant materials for several applications, such as IR technologies and laser devices [5,6]. This is because of their properties which include low maximum phonon energy (~750 cm^−1^), high refractive index (~2), good chemical stability, low melting temperature, $T_{g}$ (~350 °C), and high dielectric constant. Tellurium oxide-based glasses are known to be transparent in both visible and near IR (NIR) electromagnetic spectrum regions and to be non-hygroscopic materials [7,8,9]. The basic structural units of the tellurite glasses are trigonal bipyramidal (TeO_4_) and trigonal pyramidal (TeO_3_) units. When a modifier oxide is added into the TeO_2_ glass, it breaks the Te–O–Te linkages and forms trigonal (TeO_3_) units [10,11,12,13].

Moreover, GeO_2_ is known to be a glass former and a semiconductor material of interest [1]. Glasses based on GeO_2_ have been widely used in the fabrication of optical fibers and NIR devices because of their high refractive index and high transparency in the NIR region [14]. In addition, germanium dioxide-containing glasses are characterized by their high resistance to intense ionizing radiation and ability to absorb X-rays. These glasses have chemical durability and good thermal stability since germanium (Ge^4+^) and oxygen (O^2−^) ions have a strong interionic strength between them [13]. A special feature of glasses containing GeO_2_ and alkali metals is the germanate anomaly, and this is well known when a proportion of alkali metal (10–17.5 wt%) is introduced, and this is related to the structure of the glass. Researchers believe that saturating the interstices with alkaline ions leads to the transformation of the GeO_4_ groups into GeO_6_ groups with octahedral structures.

On the other hand, it is known that cadmium oxide (CdO) acts as a network modifier. Unlike conventional alkali or alkaline-earth oxide modifiers, cadmium oxide is thermally stable and notably covalent in character. Thus, it is expected to shorten the solidification time of glasses during the quenching process [5,13]. Depending on the concentration of CdO, the Cd^2+^ ions are probable to occupy network modifier (CdO_6_) positions, adding together to network forming locations with CdO_4_ structural units. Cadmium oxide is a chemical compound that has semiconductor and piezoelectric characteristics. Compared to other semiconductors, CdO has a gap energy of about 4.1 (eV) and higher exciton binding energy (75 meV) [15,16]. Exciton recombination of CdO nanoparticles leads to UV emissions of about 415 nm [17]. Many researchers are interested in this characteristic of the CdO used in LEDs with short wavelengths [18]. Due to the sharp exciton transition of CdO, it can be used in semiconductor lasers and as transparent electrodes of solar cells [19]. On the other, compared with nanoparticles of another toxic semiconductor, CdO nanoparticles are of the lowest toxicity [20]. Therefore, this chemical is an appropriate option for bioapplications due to its good optical characteristics, such as its fluorescence, high-resolution second-harmonic generation, and two photons’ emissions.

Previous studies of the CdO-TeO_2_-GeO_2_ system [21,22,23,24] revealed that the high content of CdO causes the formation of inverted glasses. It was also detected [21,22,23] that Cd^2+^ cations can coordinate in either tetrahedral or octahedral units; thus, it is possible that its role as a modifier can change to that of an intermediate. X-ray diffraction analysis of this ternary system showed that a sample with overall composition 30CdO·20TeO_2_·50GeO_2_ contained CdTe and AlGex nanocrystals with sizes of 59.15 nm and 28.52 nm, respectively [22]. Another study showed that glasses in the CdO-TeO_2_-GeO_2_ system were optically activated by Eu^3+^, Dy^3+^ and Dy^3+^/Eu^3+^. This vitreous system might be useful as a host for rare-earth ions, such as phosphors for UV-based white LEDs [24].

Moreover, GeO_2_ is known to be a glass former and a semiconductor material of interest [5]. Glasses based on GeO_2_ have been widely used in the fabrication of optical fibers and NIR devices because of their high refractive index and high transparency in the NIR region [14]. For the above, studies indicated that the CdO-TeO_2_-GeO_2_ system may offer the opportunity to fabricate new glasses with a variety of potential technological applications. Furthermore, the goal of this investigation was twofold: first, to produce a series of CdO-TeO_2_-GeO_2_ glasses with high content of TeO_2_ (40–80 wt%); and second, to test the effect of the amount of CdO in the formulation (10–50 wt%) on the spectroscopic properties of the glasses thus obtained.

## 2. Materials and Methods

**Figure 1:**
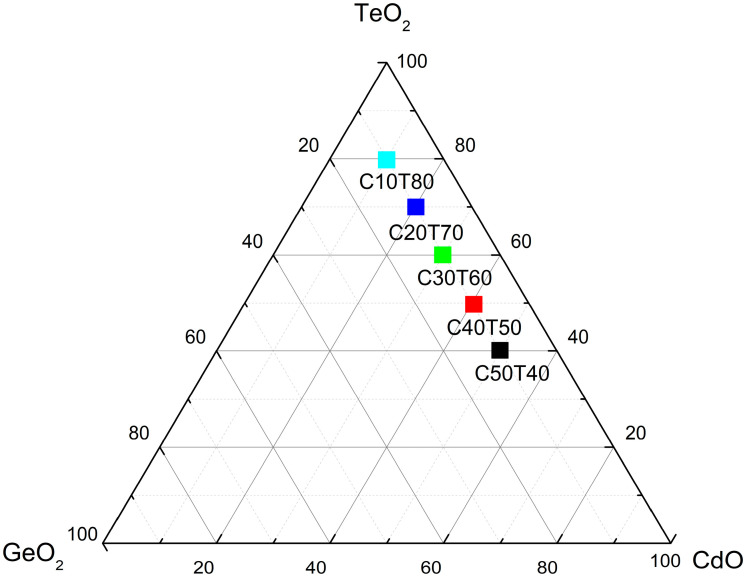
Gibbs triangle showing the compositions of the raw materials used in the present study.

The raw materials used in the present study consisted of 99.99% purity of CdO, TeO_2_ and GeO_2_ provided by Sigma Aldrich (St. Louis, MO, USA). The resulting blends may be represented by the empirical formula: xCdO-(90 − x)TeO_2_-10GeO_2_, where x indicates the content of CdO in the blend in weight percent. It is noted that the amount of GeO_2_ was set to 10% in all the blends. Five blends were prepared as shown in Figure 1, which corresponded to C10T80, C20T70, C30T60, C40T50, and C50T40. In a typical experiment, the raw materials were first mixed in a high-alumina crucible and further melted at 900 °C for 1 h in a Thermolyne furnace model 46100 (Dubuque, IA, USA). The vitrification process was further conducted by rapid cooling in a stainless-steel mold. To improve the mechanical properties, the samples were kept at 300 °C for 2 h in a stainless-steel mold. Subsequently, the oven was turned off and the samples were allowed to cool down inside the oven by natural convection.

Prior to the spectroscopic analysis, each sample was crushed and ground until the particle size was about 30 µm. Next, X-ray diffraction (XRD) analyses of the samples were conducted in a Bruker Advanced D-8 diffractometer (Madison, WI, USA), using the Cu Kα line. Raman spectra were obtained in a LabRam HR Jobin-Yvon-Horiba spectrometer (Piscataway, NJ, USA) using the He-Ne (632.8 nm) laser line. Optical absorption (OA) spectra were measured in a Varian (Agilent, Santa Clara, CA, USA) spectrometer, model CARY 5000. The optical band gap was calculated assuming an indirect band gap by using the Tauc method, since these materials are amorphous [25]. XPS spectra were obtained in a PHI 5100 spectrometer (Perkin-Elmer, Waltham, MA, USA) with a Mg X-ray Kα radiation of 1253.6 keV, with a pass energy of 20 eV for high-resolution spectra of C 1s, O 1s, Ge 3d, Cd 3d, and Te 3d core levels, using as reference the C 1s peak at 284.8 eV.

## 3. Results and Discussion

### 3.1. X-Ray Diffraction Analysis

**Figure 2:**
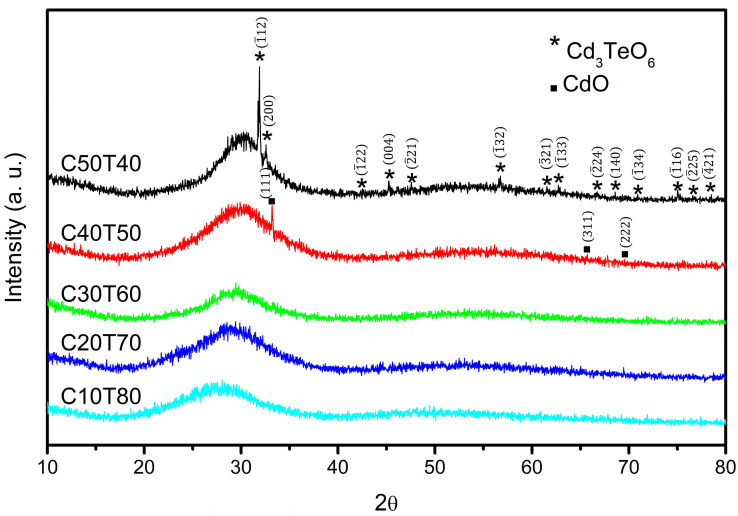
X-ray diffraction patterns of samples.

Under visual inspection, the glasses obtained in this work showed a homogeneous appearance. The samples changed from colorless to clear green, green, and brown-orange as the amount of CdO was varied. X-ray diffraction patterns of the samples are shown in Figure 2. All glasses showed a broad diffraction band, in which the maximum as a function of 2θ shifted from 30° to 28° as the amount of TeO_2_ was increased. The broad band indicates a disordered structure of large range. However, the increase in the concentration of modifier oxides such as CdO in the TeO_2_ network causes a change in the coordination of tellurium, from TeO_4_ (tbp) to TeO_3_ (tp) [26]. This transformation may be associated with an increase in non-bridging oxygens (NBOs) and the development of crystalline phases.

Sample C50T40 exhibited diffraction peaks corresponding to nanocrystals of the Cd_3_TeO_6_ phase, with lattice parameters a = 5.4986 Å, b = 5.6386 Å, and c = 8.0191 Å. The diffraction peaks at 2θ values of 31.89°, 32.56°, 42.50°, 45.25°, 47.57°, 56.76°, 61.50°, 62.71°, 66.62°, 68.50°, 70.92°, 75.09°, 76.58°, and 78.48° are associated with the monoclinic structure (JCPDS 76-1007 [27]). Sample C40T50 showed peaks corresponding to nanocrystals of the CdO phase, with a lattice parameter of a = 4.69485 Å. The diffraction peaks at 33.18°, 65.69°, and 69.53° confirm its cubic structure (JCPDS 75-0591 [28]). The crystal sizes calculated by means of the Scherrer’s equation were found to be 84 and 93 nm for Cd_3_TeO_6_ and CdO phases, respectively.

### 3.2. Raman Spectroscopy

**Figure 3:**
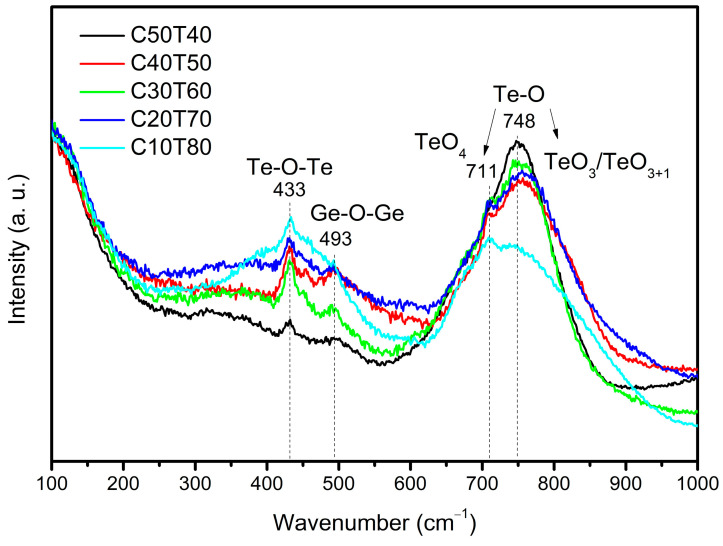
Raman spectra of samples C50T40-C10T80 showing the low and high frequency regions as discussed in the text.

The evolution of the glass structure with the addition of the modifier can be inferred from the Raman analysis results shown in Figure 3. In general, the spectra can be grouped into two regions: the low-frequency region (237–570 cm^−1^) and the high-frequency region (600–1000 cm^−1^). In the low-frequency region, two predominant bands centered at 433 and 493 cm^−1^ marked with dashed line are observed. The band located at 433 cm^−1^ corresponds to the symmetrical stretching or bending vibrations of Te-O-Te linkages at corner-sharing sites. This mode is due to the networking of the glass structure. Because the intensity of this band gradually decreased as the amount of CdO was increased, this indicates the loss of connectivity upon the addition of the glass modifier. The band at 493 cm^−1^ can be related to the vibration of Ge(IV)-O-Ge(IV) bridges in three-membered rings formed from tetrahedra [GeO_4_] [5,29,30]. This band increases in intensity without a linear relationship with the TeO_2_ content; however, the band disappears for the sample with a higher TeO_2_ concentration because for this sample, the Te-O-Te bonds predominate, as can be seen in the Figure 3.

In the high-frequency region, the intensity of the main band was found to be of similar magnitude in samples C50T40, C30T60, C40T50, and C20T70. However, it was substantially lower in sample C10T80. The band at 711 cm^−1^ appears as a small peak over a broad band located at 748 cm^−1^. At this position, asymmetric stretching vibrations of Te-O- bonds in the TeO_4_ bipyramidal structural units occur [31]. The band at 748 cm^−1^ is the most intense band in all samples. This band is attributed to the stretching modes of Te-O- and Te=O bonds, which contain non-bridging oxygen (NBO) from TeO_3_ tp and TeO_3+1_ polyhedra [5,32]. This band is typically undetected in pure TeO_2_. The addition of glass modifiers results in the cleavage of the Te-O-Te linkage of initially completely polymerized structure and transforms TeO_4_ units into TeO_3+1_ polyhedra having one NBO atom or TeO_3_ tp with more NBO atoms [13].

Thus, the observed shift indicates a structure evolution from a rich TeO_2_ phase to Cd_x_Te_y_O_z_ compounds that contain TeO_3_ structures [33,34]. The consumption of the TeO_2_ phase and the formation of Cd^+2^-(TeO_3_)_n_ phases is also observed in their respective XRD patterns.

### 3.3. Raman Deconvolution

**Figure 4:**
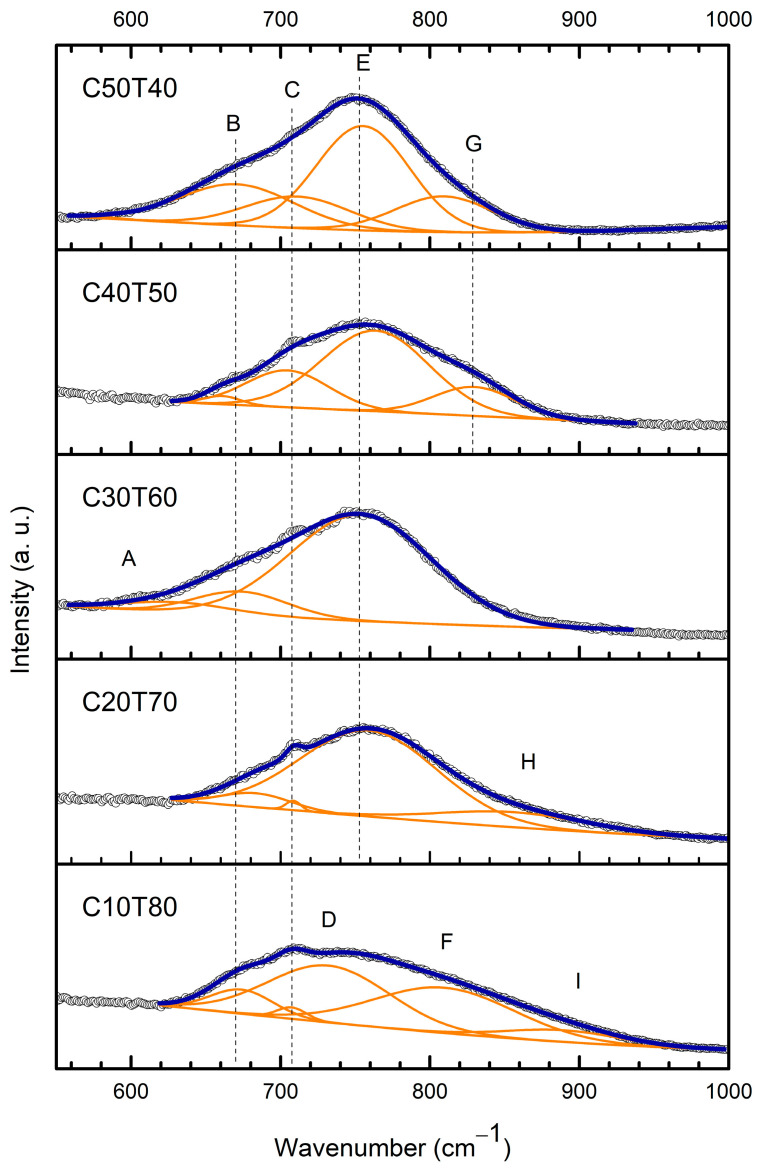
Deconvolution Raman spectra of C50T40-C10T80 high frequency regions.

Figure 4 shows the deconvoluted Raman spectra of all samples which were background-corrected and Gaussian functions were used to decompose. It is noted that the main band in the 1000–600 cm^−1^ range is made up of the contributions of nine individual bands designated by symbols A through I in the plots. The intensity of the individual bands in the samples depended on the amounts of CdO and TeO_2_ contained in the samples.

The analysis was conducted in the range of 1000–600 cm^−1^, and the results are listed in Table 1. In general, the intensity of the deconvoluted band increased as the content of TeO_2_ decreased. The small band A at 621–630 cm^−1^ corresponds to the O-Te-O in the TeO_4_ unit and to ring strain associated with the bending modes of Ge-O-Ge bonds [35,36]. Band A was only detected in sample C30T60. Band B is related to the stretching modes of O-Te-O bonds in TeO_4_ units. It can be associated with antisymmetric vibrations of Te-O bonds in the TeO_4_, TeO_3+1_, and TeO_3_ units [32,37,38,39,40]. It is noted that the calculated intensity of band B at 661–683 cm^−1^ varied in all samples. Thus, in the sample C40T50, it was substantially low and shifted to a shorter wavelength of 660 cm^−1^. In contrast, in sample C50T40, the intensity of band B reached its highest value. Furthermore, as the amount of modifier oxide was increased, the intensity of band B also increased.

Band C at 705–709 cm^−1^ was detected in all samples except C30T60. In the samples where it was predicted to be present, its intensity varied significantly. This was likely attributed to the relative amounts of the modifier oxide. Band C can be related to the deformation modes in germanium atoms in the glassy network and to the stretching vibrations of TeO_3+1_ [37,41].

Band D at 732 cm^−1^ was found to be present only in sample C10T80. This band is associated with stretching vibrations between tellurium and non-bridging oxygen (NBO) atoms [37,41].

Band E at 754–764 cm^−1^ was found to be the most intense of all individual bands detected. It was predicted to occur in all samples except C10T80. This band is associated with asymmetric vibrations from Te-O-Te bridges in γ-TeO_2_ and stretching vibrations from groups TeO_3+1_ and [TeO_3_]^−2^ with three terminal oxygens [5,42,43,44,45,46]. It is noted that γ-TeO_2_ is a metastable TeO_2_ polymorph that arises from the rearrangement of the glass structures and eventually leads to the formation of α-TeO_2_ [47,48]. In the samples prepared in this work, the γ-TeO_2_ network constituted by polymerized TeO_4_ units connected with TeO_3_ groups was maintained, and no bands related to α-TeO_2_ were identified [47,48,49]. The former is related to the breaking of the connected glass network, whereas the latter is associated with TeO_3_ groups. This interaction caused the Te-O- bond stretching vibration studied by Sekiya et al. [40].

Band F at 810 cm^−1^ was found to be present in sample C10T80 only. This was the sample with the highest content of the former oxide. This band can be associated to γ-TeO_2_; short Te-O- bonds with NBOs form TeO_4_ units. It is also related to the LO split of the antisymmetric stretching vibration into the Q^2^ unit [32,35,36,50].

Band G at 830 cm^−1^ was found to be present in samples C40T50 and C50T40 which contained the lowest amounts of TeO_2_. It may be related with the asymmetric stretching of Ge-O-Ge bridges in GeO_4_ tetrahedra [41]. Band H at 856 cm^−1^ was only observed in sample C20T70 and can be associated with the stretching vibrations of NBO in Q^3^ species (GeO_4_ with three bridging oxygen atoms) [41,51].

Finally, band I at 889 cm^−1^ was characteristic of the sample with the highest TeO_2_ content C10T80 and may be attributed to the vibrations of TeO_4_ tbp and the Te-_eq_O_ax_-Te bond. Moreover, band I may correspond to the transverse optic (TO) asymmetric stretching vibration of bridging oxygen in Ge-O-Ge linkage in GeO_4_ units [35].

Overall, it is known that the intensities and locations of the Raman bands detected in each sample depend on both the concentration and types of the structural groups in the sample. It is noted that the individual bands in the deconvolution results vary in intensity and some of them shift towards longer wavelengths. This behavior was possibly caused by the presence of the modifying oxide, the formation of GeO_4_ units, and the replacement of some TeO_3+1_ and TeO_3_ units by GeO_4_ units [52,53].

**Table 1:** Assignment of component bands in deconvoluted Raman spectra.

Band Label	Location (cm^−1^)	Band Assignment	References
A	621–630	Stretching modes of O-Te-O in TeO_4_ tbp units, and/or ring strain from Ge-O-Ge bending vibrations.	[35,36]
B	661–683	Stretching modes of O-Te-O in TeO_4_ tbp units, antisymmetric vibrations of Te-O bonds in TeO_4_, TeO_3+1_ and TeO_3_ units.	[32,37,38,39,40]
C	705–709	Assigned to the deformation modes of Ge atoms in the glassy network.	[37,41]
D	732	Stretching vibration between tellurium and non-bridging oxygen (NBO) atoms.	[37,41]
E	754–764	Asymmetric vibrations of Te-O-Te symmetric bridges in γ-TeO_2_, stretching vibrations from groups TeO_3+1_ and [TeO_3_]^2−^ with three terminal oxygens.	[5,35,42,43,46,54]
		The peaks around 713 and 747 are assigned to stretching vibrations of TeO_3_/TeO_3+1_ unit.	[42]
F	810	Characteristic band of γ-TeO_2_, short Te-O- bonds with NBOs form TeO_4_ units; LO split of the antisymmetric stretching vibrations into Q^2^ units.	[32,35,36,50]
G	830	Could arise from that in Ge Q^3^-species.	[41]
H	856	Assigned to the stretching vibrations of NBO in Q^3^ species (GeO_4_ with three bridging oxygen atoms).	[41,51]
I	889	Stretching vibrations from Te=O in TeO_3_ isolated units, TO split of the antisymmetric stretching vibrations of Ge-O-Ge in Q^3^ units.	[35]

### 3.4. XPS Analysis

**Figure 5:**
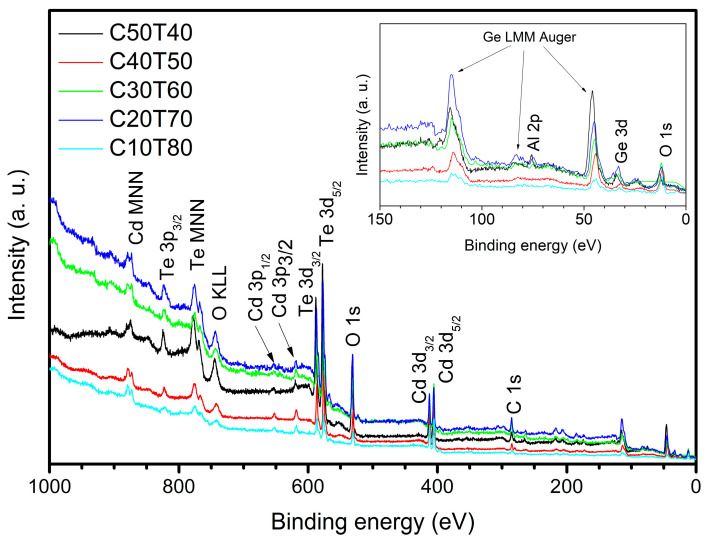
XPS scan of fabricated glasses presenting the photoelectron peaks related to the elements that constitute the glasses; the inset shows the region from 150 to 0 eV where the Ge 3d peak appears as well as the Al 2p for some samples.

Based on XPS wide scans of the samples, the characteristic photoelectron peaks of Cd, Ge, Te, and O were identified in the samples; this is shown in Figure 5. In samples C50T40, C30T60, and C20T70, some Al was also detected. This element was most likely incorporated into the glass because of crucble corrosion (see inset in Figure 5). In general, the introduction of crucible material depends on melting temperature and time [55]. However, all samples were obtained under the same conditions of melting temperature and time. The composition between samples varies, and therefore, there may be more crucible corrosion in some samples than in others because an uncontrolled amount of crucible material, which can be transferred to the melt, significantly depends on the glass composition and temperature–time conditions of its melting [56].

**Figure 6:**
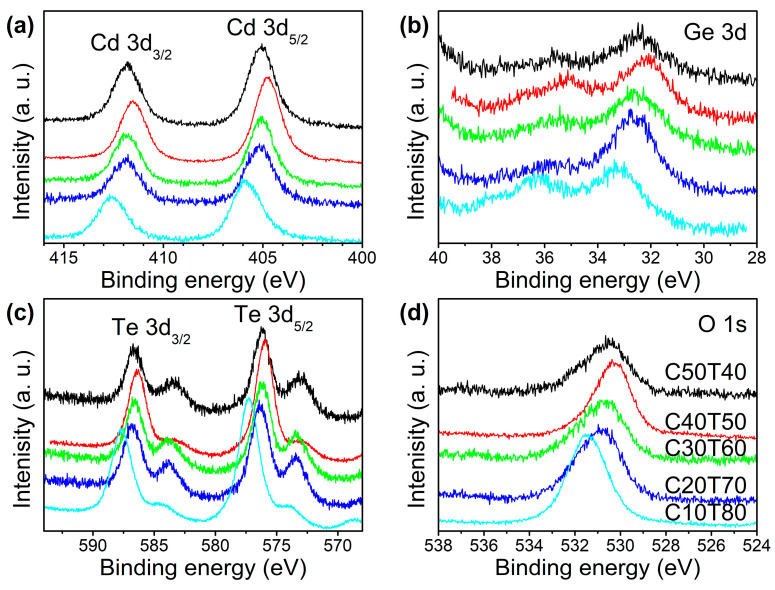
High-resolution XPS spectra of (**a**) Cd 3d_5/2_ and Cd 3d_3/2_, (**b**) Ge 3d, and (**c**) Te 3d_5/2_ and Te 3d_3/2_; and (**d**) O 1s photoelectron peaks of all glass samples.

**Figure 7:**
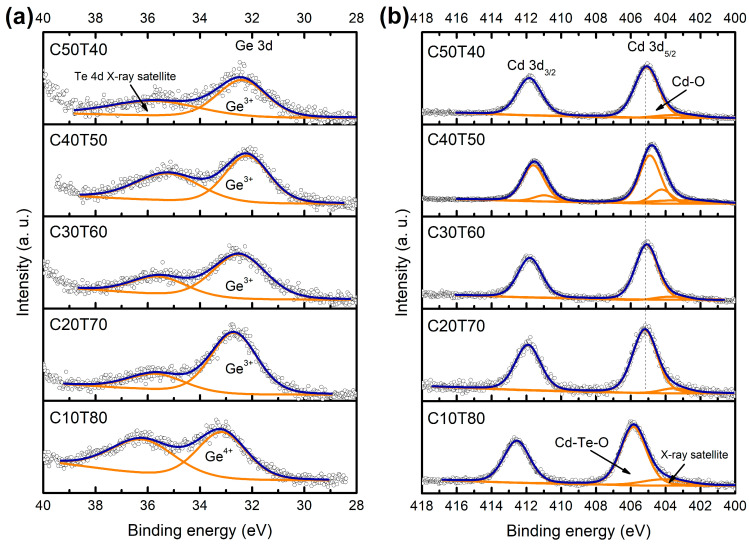
Curve fitting of (**a**) Ge 3d and (**b**) Cd 3d doublet high-resolution spectra.

**Figure 8:**
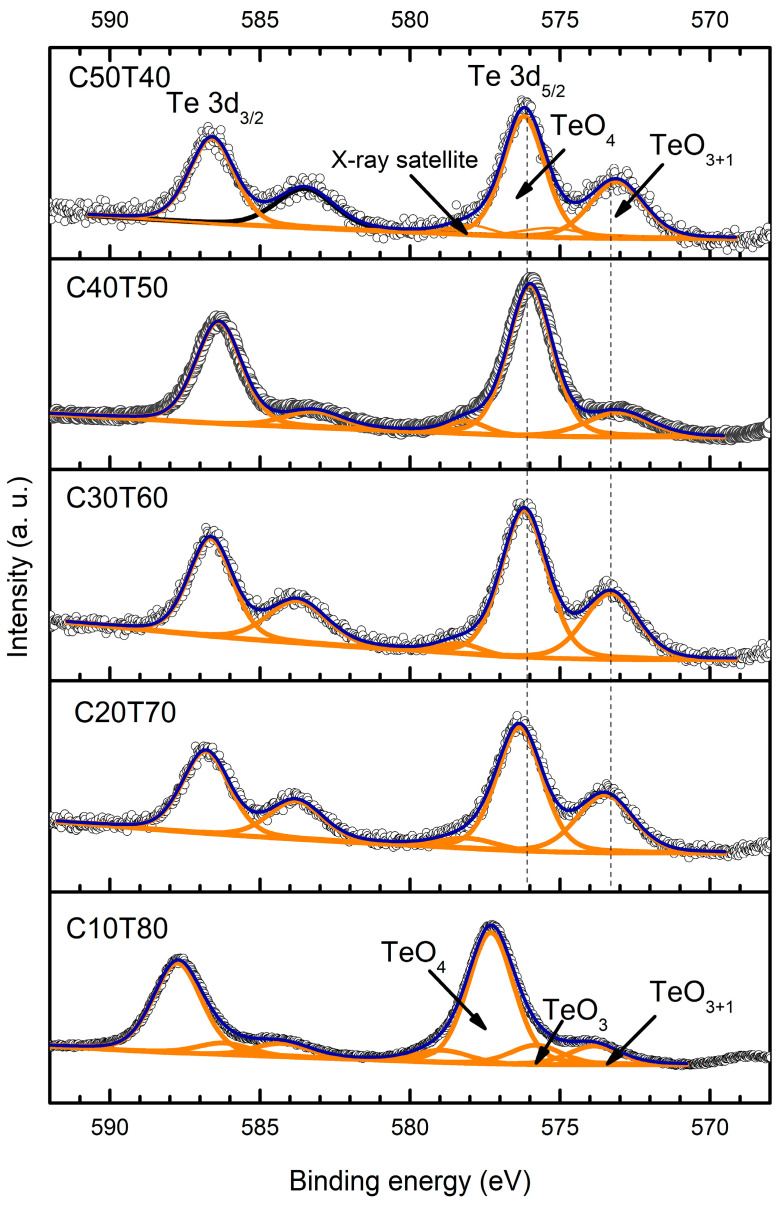
Curve fitting of Te 3d.

To follow up the structural evolution of the glasses as a function of the initial amounts of GeO_2_, CdO, and TeO_2_, high-resolution spectra of Cd 3d_5/2_, Cd 3d_3/2_, Ge 3d, Te 3d_3/2_ Te 3d_5/2_, and O 1s were conducted in all samples. The results are shown in Figure 6a–d. It is noted that samples C50T40, C30T60, and C20T70 showed similar spectra. Sample C40T50 showed a slight shift towards low binding energy values, and sample C10T80 shifted to higher binding energy values. For a further analysis, deconvolution of all high-resolution spectra (Figure 7 and Figure 8) was carried out using a Gaussian–Lorentzian function and a Tougaard background subtraction. The results are shown in Table 2.

The curve fittings of the Ge 3d photoelectron line of all samples are shown in Figure 7a. A shoulder at energy values around 35.6 eV may be the X-ray satellite of the Te 4d [57,58]. The peaks of the 3d Ge for glasses C50T40–C20T70 were in the range of 32.2 to 32.7 eV. They are related to Ge^3+^ in Ge_2_O_3_ [59]. Regarding sample C10T80, the position of the peak shifted towards a higher binding energy (33.2 eV), and the oxidation state of germanium changed to Ge^4+^ (GeO_2_) [59,60,61]. It is noted that the high content of TeO_2_ changed the coordination of Ge. Thus, four oxygen atoms remove the electronic density from the germanium atom. A possible explanation is that there are GeO_4_ units with two or more NBOs. The electron density is polarized toward the oxygen atom, decreasing the charge density around the Ge atom, which results in the 3d photoelectronic peak shifting toward higher binding energy values. Considering that the GeO_2_ content does not vary among the different glasses’ compositions, Ge-O-Te bonds may form in glasses with high TeO_2_ content.

XPS spectra of Cd 3d are shown in Figure 7b. The binding energies of 405.2, 404.2, 404.9, 405.1, 405.2, and 405.85, eV were obtained for 3d_5/2_. Similarly, the values of 411.9, 410.9, 411.6, 411.8, and 412.6 eV, were obtained for 3d_3/2_. The binding energy is attributed to the Cd^2+^ bonding state [62], shifted to lower binding energy values regarding Cd-O bonds. For C50T40 glass, the Cd 3d_5/2_ peak is observed at 405.2 eV, which is commonly related to Cd-Te in cadmium telluride [63,64]. According to XRD results, this sample exhibits the formation of tricadmium orthotellurate crystals. Since the 3d doublet could not be resolved in more components, it is possible to infer that there is a combination of Cd-O bonds from the crystal CdO_6_ octahedra [65] and Cd-O bonds in a TeO_2_-rich matrix that causes the shift to higher binding energies. Furthermore, the effect of cadmium oxide nanocrystals on the behavior of sample C40T50 is noted. Figure 7b shows that the most intense band of the Cd 3d_5/2_ peak shifted to the right, and the less intense band of that same peak shifted to the left. The concentration of Cd^2+^ ions within the glass matrix decreases, acting as a modifier, and interacts with two Te-O- terminal bonds, thereby decreasing the local electron density of Cd, causing the shift to 405.85 eV [62,66]. Another possibility is the formation of Cd-O-Te bonds, which present low local electron densities, leading to higher binding energy values for Cd^2+^ ion photoelectrons. It is necessary to analyze this system further to understand its glass network structure.

Deconvoluted Te 3d XPS spectra are shown in Figure 8. The Te 3d spectra are associated with different tellurium-based structural units such as TeO_4_, TeO_3_, and TeO_3+1_ [67]. Such associations were made based on electronegativity and binding energy considerations [68]. Thus, the Te 3d_5/2_ peak at 573–573.85 eV was assigned to Te^0^, and the 575.8–576.3 eV peak was associated with Te-O links within the TeO_2_ network [69,70,71]. However, it is noted that the band position in sample C10T80 changed to 577.15 eV, which suggests a change in the coordination of TeO_2_, possibly from TeO_3_ to TeO_4_ [67,72,73].

On the other hand, the characteristic Te 3d_5/2_ photoelectron peak shifts to higher energy values in the range of 576–577.3 eV as the TeO_2_ content increases. Based on an extensive bibliographic review, tellurium is present as Te^4+^ in TeO_4_ groups for the 576.2 eV, 576 eV, 576.2 eV, 576.3 eV, and 577.3 eV peaks, its Te 3d_3/2_ counterparts (Table 2) separated by ~10.4 eV [67,72,73,74,75]. The peak at lower binding energies in the 573–573.85 eV range can arise from tellurium in TeO_3+1_ units [67,73]. Additionally, the FWHM did not change drastically from ~2.1, except for in the C40T50 sample, which had an FWHM of 2.53. The sample presented a CdO crystalline phase, which translates into a TeO_2_ enrichment of the glass matrix. According to previous reports, the Te 3d_5/2_ peak related to the TeO_3_ trigonal pyramids appears around 575 to 576 eV [67,72,73]. Then, for sample C10T80, a peak at 575.8 eV can be assigned to Te^4+^ in TeO_3_ groups, and the peak at 577.3 eV is attributed to TeO_4_ or Te in Ge-O-Te bonds. Those results are in agreement with the Raman spectra analysis. Moreover, the formation of metallic Te cannot be discarded. P. Wang et al. [76] performed an analysis of the effect of melting temperature on color-changing in TeO-GeO-K_2_O-Nb_2_O_5_ glasses using alumina crucibles for the fusion, confirming the presence of metallic Te with differential scanning calorimetry measurements. However, they did not perform XPS spectra deconvolution of the Te 3d doublet peaks, although a shift in position was evidenced. M. Xu et al. [74] reported that for Te^0^ nanostructures, the Te 3d_5/2_ and 3d_3/2_ peaks appear at 573 eV and 583 eV, respectively. For samples C50T40 and C40T50, the Te 3d doublet peaks have binding energies close to those of metallic Te; however, this cannot be confirmed with the present analysis. It is similar to the formation of Cd-Te bonds. According to XPS data on CdTe, the peaks exhibit binding energies of 570–572.5 eV and 581–582 eV [77,78,79], which are lower than those found in the analyzed glasses.

**Figure 9:**
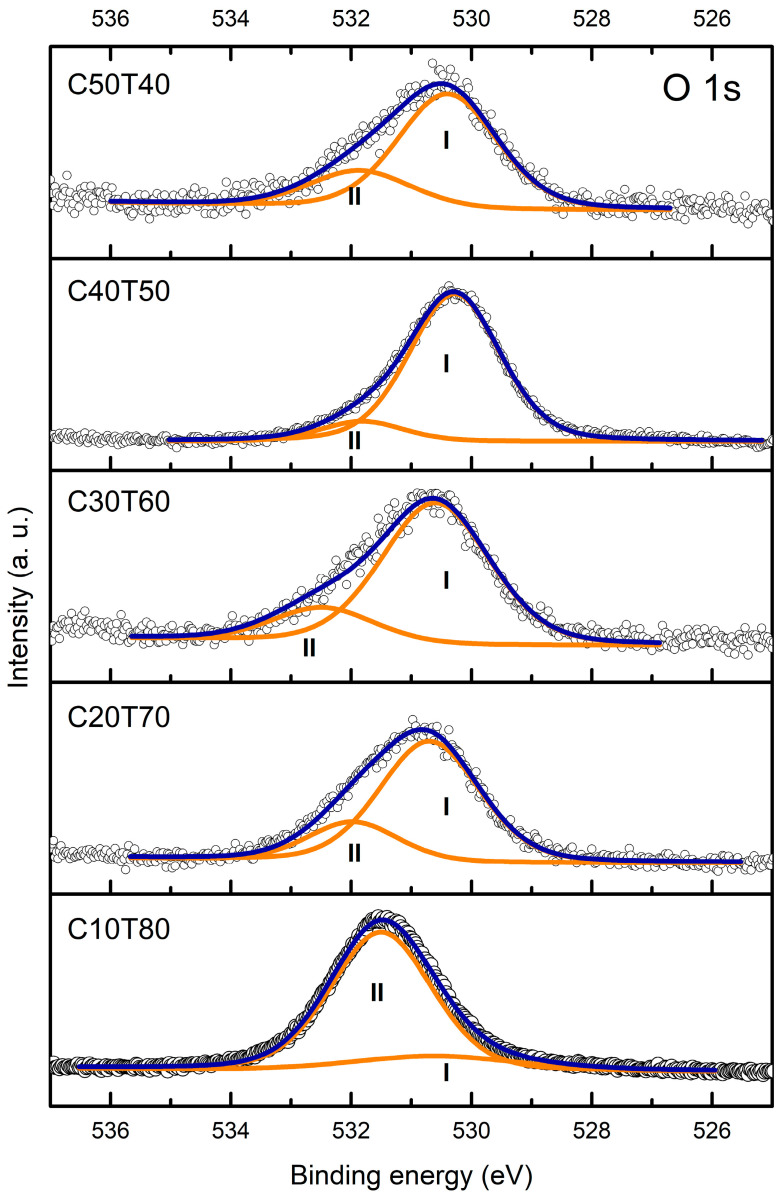
O 1s peaks of all samples.

The peaks due to non-bridging oxygen atoms (NBOs) and the bridging oxygen atoms (BOs) (Table 3) were separated by deconvoluting the O 1s spectrum (Figure 9) into different Gaussian peaks. The O 1s (I) denotes the NBOs, and O 1s (II) represents the BOs with lower and higher binding energy values, respectively [65,72,73]. In samples C50T40 and C40T50, the NBOs’ peak position is almost the same, but the area increases significantly for the C40T50 glass. However, the Te 3d spectra showed that the TeO_4_ units are the most intense for that glass composition. It is necessary to consider that Ge-O^−^, Te=O, Te-O^−^, and Cd-O bonds contribute to the NBOs’ peak, not just TeO_3_ groups [72]. For samples C30T60, C20T70, and C10T80, the value of the O_I_ peak shifts to ~530.6 eV and decreases its area proportion gradually until it collapses for the glass C10T80. This change arises from the formation of TeO_4_ units, as discussed in the analysis of the Te 3d XPS spectra. Meanwhile, the O_II_ peak from BOs appears in the 531.8 eV to 532.5 eV range, with the sample of C30T60 glass exhibiting the highest BO peak value, contributing only 11% to the O 1s peak area. This is interesting because despite the high content of TeO_2_, it is observed that only a few Te-O-Te bonds contribute. From the Raman spectroscopy analysis of the C30T60 glass, we can infer that the NBOs predominantly arose from [TeO_3_]^2−^ units. This means that the network connectivity may be facilitated by a few TeO_4_, GeO_2_, and CdO units, as the last oxide has demonstrated the capability of being a glass former [21] or through Te-O-Cd and Te-O-Ge bonds that do not contribute to the BOs’ peak. Additionally, samples C50T40, C30T60, and C20T70 exhibited and displayed evident Al incorporation (Figure 5), which forms Te-O-Al bonds that also contribute to network connectivity. In addition, XRD characterization evidenced that those three glasses were entirely amorphous. For the C10T80 glass, there was a drastic increase in the BOs’ peak area contribution, with a FWHM of 3.23, which is consistent with the appearance of TeO_4_ units, as evidenced by the Te 3d spectra of this sample.

Finally, it was previously reported that in TeO_2_ glasses, the BO and NBO components cannot be resolved due to the initial distorted symmetry of TeO_2_ structural units [80]. Consequently, the peak BOs at higher energy can be assigned to Te-O-Te and Ge-O-Ge, and the peak NBOs at lower energy values may be associated with Ge-O-Cd, Te-O-Cd, and Cd-O bonds.

### 3.5. Optical Absorption

**Figure 10:**
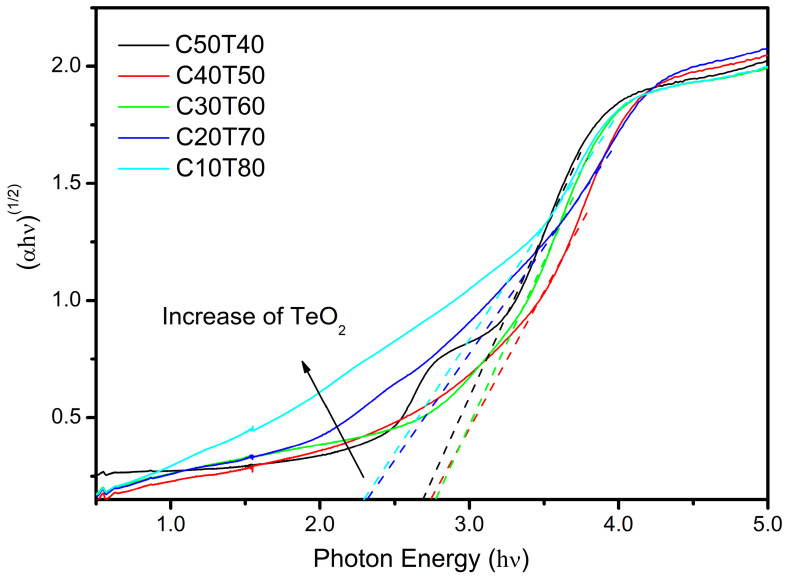
Plot of (hνα)1/2 versus photon energy (hν) for the samples C50T40-C10T80, as is indicated. The straight line segment represents the best linear fitting of the absorption edge to the energy axis.

The results of the optical absorption analysis of the samples are shown in Figure 10. By means of the Tauc’s relation, a plot of $\left(h\nu \alpha )n\;vs.\;(h\nu -E_{g}\right)$ can be used to calculate the optical band gap $\left(E_{g}\right)$. In this relationship, α is the absorption coefficient, h is the Planck’s constant, *ν* is the frequency of the electromagnetic radiation, and n=1/2 for amorphous materials [81].

Moreover, Table 4 shows the calculated $E_{g}$ values for all samples obtained in this work. The band gap values indicate that these glasses may have semiconductor applications [82]. It is noted that the larger the content of TeO_2_, the lower the value of $E_{g}$. This behavior may be attributed to the effect of the bridging oxygen linkages in the structure. A distinctive feature is noted in sample C50T40, as two optical shoulders are observed in Figure 10. The first shoulder (3.5–4.0 photon eV) is associated with the amorphous matrix, whereas the second shoulder (2.6–3.2 eV) may be related to the effect of embedded nanoparticles of Cd_3_TeO_6_ in the glass structure detected in the XRD analysis (Figure 2).

This sample, C50T40, corresponds to a very particular composition where the modifying oxide is present in the same proportion as the forming oxide. If we observe the Raman spectra, we can see the most intense band corresponding to the formation of TeO_3_ and TeO_3+1_ units, which lead to the formation of non-bridging oxygens (NBOs). NBOs are formed by the addition of a modifying oxide, which causes a loss of lattice connectivity, giving rise to the formation of the vibrational modes Te-O-Cd and Te-O-Ge because the electrons in NBOs are more easily excited because they are weakly bound compared to bridging oxygens (BOs) [83].

The band gap varies with the presence of non-bridging oxygens. On the other hand, as the amount of CdO (10–40% wt) increases, the value of $E_{g}$ also increases, and it would be expected that at high concentrations of NBOs, the band gap energy would be narrower. In the glasses under study, this does not happen; they exhibited different behavior, perhaps due to the dual role of cadmium, by coordinating with the TeO_4_ and GeO_2_ groups.

### 3.6. Refractive Index

The refractive index (n) is one of the key properties to be determined in a glass. This is because the refractive index affects the magnitude of the speed of light through the glass matrix and its direction upon being refracted. The empirical relationship between $E_{g}$ and n is: $n^{2}={\left(180/E_{g}\right)}^{1/2}-2$ [84]. By using the values of $E_{g}$ computed in the previous section, the values of n shown in Table 4 were obtained. Overall, the refractive index varied in the range of 2.432–2.609, and it increased as the amount of TeO_2_ increased in the samples. Glasses with similar refractive index have been suggested in applications of waveguide in the sub-THz and millimeters wave region [85]. Typically, an increase in the value of n has been associated with an increase in the polarizability of the matrix, which in turn is caused by the growth of the population of NBOs that possess a greater number of polarizabilities compared to bridging oxygen [84,85,86]. In the present case, in addition to the increase in NBOs, a large number of Cd^2+^ ions were incorporated into the glassy matrix. Such Cd^2+^ ions possess large polarizability and a high coordination number. In general, the present glasses show potential for applications in optical devices and fiber optics. The TeO_2_–GeO_2_ combination allowed the fabrication of glass samples with high values of the refractive index, high light transmission, and selective absorption compared to those of borosilicate and silicate glasses.

## 4. Conclusions

Glasses with high TeO_2_ content (40–80 wt%) were successfully obtained by the traditional melt-quenching process at 900 °C.

The amorphous nature was confirmed by XRD; however, crystallization of the CdO-rich samples occurred and the presence of nanocrystals was observed. Cadmium orthotellurate (Cd_3_TeO_6_) nanocrystals were obtained in the C50T40 glass, and in the C40T50 glass, cadmium oxide nanocrystals were detected. So, one of the effects of CdO in these glasses is that high quantities produce a nanocrystalline embedded in the glass phase.

Raman spectroscopy analysis results revealed the transformation of structural units from TeO_4_ → TeO_3_ in the glass system with the addition of CdO (modifier oxide). It also showed the presence of M-O-M bonds and higher disorder in the glass network when the TeO_2_ content was larger than 60 wt%.

The XPS analysis showed that as the content of TeO_2_ increases, there is a shift towards higher binding energies of the characteristic peaks of Cd 3d_5/2_, Cd 3d_3/2_, Ge 3d, Te 3d_3/2_, Te 3d_5/2_, and O 1s.

The band gap energy was reduced from 2.87 to 2.32 eV. This behavior was mainly associated with the increase in polyhedron networks induced by M-O-M (M = Cd, Te) chemical bonds, promoting sp transitions located at longer wavelengths.

These glasses can be suitable for optical amplifiers and non-linear optical materials used in the field of science and engineering.

## Figures and Tables

**Table 2:** Results from curve fitting of high-resolution XPS spectra of Ge 3d, Cd 3d_5/2_, Cd 3d_3/2_, Te 3d_5/2_, and Te 3d_3/2_ peaks.

Glass	Ge 3d		Cd				Te			
			3d_5/2_		3d_3/2_		3d_5/2_		3d_3/2_	
	eV	FWHM	eV	FWHM	eV	FWHM	eV	FWHM	eV	FWHM
C50T40	32.4	2.24	405.2	1.65	411.9	1.70	573.1	2.13	583.5	2.28
							576.2	1.65	586.6	1.79
C40T50	32.2	1.97	404.2	1.37	410.9	1.44	573.0	253	583.2	2.26
			404.9	1.42	411.6	1.49	576	1.75	586.4	1.80
C30T60	32.5	2.44	405.1	1.51	411.8	1.6	573.3	2.13	583.75	2.31
							576.2	1.81	586.6	1.72
C20T70	32.7	2.08	405.2	1.66	411.9	1.71	573.5	2.13	583.8	2.12
							576.3	1.78	586.8	1.85
C10T80	33.2	5	405.85	1.75	412.6	1.71	573.85	2.02	584.25	2.21
							575.8	2.02	586.2	2.10
							577.3	1.80	587.75	1.92

**Table 3:** Results from O 1s curve fitting of all glasses.

Glass	Component	Binding Energy (eV)	FWHM (eV)	Area (%)
C50T40	O_I_ O_II_	530.4 531.9	1.96 1.97	75.9 24.1
C40T50	O_I_ O_II_	530.3 531.8	1.78 1.63	89.0 11.0
C30T60	O_I_ O_II_	530.6 532.5	2.13 2.03	80.7 19.3
C20T70	O_I_ O_II_	530.7 532.0	1.96 1.78	76.9 23.1
C10T80	O_I_ O_II_	530.6 531.5	1.99 3.23	15.0 85.0

**Table 4:** Optical band gap and refractive index values obtained for the glass samples.

Sample	$E_{g}$ (eV)	n
C50T40	2.68	2.489
C40T50	2.87	2.432
C30T60	2.77	2.461
C20T70	2.33	2.605
C10T80	2.32	2.609

## Data Availability

The original contributions presented in this study are included in the article. Further inquiries can be directed to the corresponding authors.

## Abbreviations

XPS	X-ray photoelectron spectroscopy
IR	Infrared
NIR	Near Infrared
UV	Ultraviolet
LEDs	Light-Emitting Diodes
XRD	X-ray diffraction
OA	Optical absorption
NBOs	Non-bridging oxygen atoms
FWHM	Full Width at Half Maximum
BOs	Bridging oxygen atoms
